# Engineering *Camelina sativa* (L.) Crantz for enhanced oil and seed yields by combining diacylglycerol acyltransferase1 and glycerol‐3‐phosphate dehydrogenase expression

**DOI:** 10.1111/pbi.12847

**Published:** 2017-11-19

**Authors:** Sudesh Chhikara, Hesham M. Abdullah, Parisa Akbari, Danny Schnell, Om Parkash Dhankher

**Affiliations:** ^1^ Stockbridge School of Agriculture University of Massachusetts Amherst Amherst MA USA; ^2^ Biotechnology Department Faculty of Agriculture Al‐Azhar University Cairo Egypt; ^3^ Department of Plant Biology Michigan State University East Lansing MI USA; ^4^ Present address: Centre for Biotechnology Maharshi Dayanand University Rohtak 124001 India

**Keywords:** *Camelina sativa*, triacylglycerols, biofuels, lipid metabolism, metabolic engineering

## Abstract

Plant seed oil‐based liquid transportation fuels (i.e., biodiesel and green diesel) have tremendous potential as environmentally, economically and technologically feasible alternatives to petroleum‐derived fuels. Due to their nutritional and industrial importance, one of the major objectives is to increase the seed yield and oil production of oilseed crops via biotechnological approaches. *Camelina sativa*, an emerging oilseed crop, has been proposed as an ideal crop for biodiesel and bioproduct applications. Further increase in seed oil yield by increasing the flux of carbon from increased photosynthesis into triacylglycerol (TAG) synthesis will make this crop more profitable. To increase the oil yield, we engineered Camelina by co‐expressing the *Arabidopsis thaliana* (L.) Heynh. *diacylglycerol acyltransferase1* (*DGAT1*) and a yeast cytosolic *glycerol‐3‐phosphate dehydrogenase* (*GPD1*) genes under the control of seed‐specific promoters. Plants co‐expressing DGAT1 and GPD1 exhibited up to 13% higher seed oil content and up to 52% increase in seed mass compared to wild‐type plants. Further, DGAT1‐ and GDP1‐co‐expressing lines showed significantly higher seed and oil yields on a dry weight basis than the wild‐type controls or plants expressing DGAT1 and GPD1 alone. The oil harvest index (g oil per g total dry matter) for DGTA1‐ and GPD1‐co‐expressing lines was almost twofold higher as compared to wild type and the lines expressing DGAT1 and GPD1 alone. Therefore, combining the overexpression of TAG biosynthetic genes, *DGAT1* and *GPD1*, appears to be a positive strategy to achieve a synergistic effect on the flux through the TAG synthesis pathway, and thereby further increase the oil yield.

## Introduction

Vegetable oils, composed mainly of triacylglycerols (TAGs), are important nutritional and industrial commodities. They can provide the nutritional requirements of humans and animals and provide chemical feedstocks involved in various industrial products, including biofuels (van Erp *et al*., 2014; Vigeolas *et al*., 2007). The worldwide production of vegetable oils has increased dramatically in recent decades, and according to the last statistics of year 2015/2016, it is approximately 179.5 million metric tons (MMT) per year, with the majority produced from palm (65.5 MMT), soybean (53.7 MMT), rapeseed (26.6 MMT, The statistics portal, Statista, www.statista.com). Despite this increased production of vegetable oils, a wider gap between the production and consumption exists. To overcome the societies’ ever‐growing demands for vegetable oils, there is considerable interest in the metabolic engineering of improved seed oil yields and qualities (van Erp *et al*., 2014).

The application of genetic engineering approaches to boost the metabolic flux of carbon into seed oils has been limited mainly by the benightedness of how lipid metabolism is regulated (Taylor *et al*., 2009). The pathway for the metabolic flux of carbon into seed storage lipids is complex and a multigene process and involves various subcellular compartments (Durrett *et al*., 2008; van Erp *et al*., 2014; Schwender *et al*., 2004). Briefly, in the developing seeds, sucrose (Suc) is unloaded in the phloem and metabolized into glucose‐6‐phosphate (G6P) via either Suc synthase‐ or invertase‐dependent pathways (Barratt *et al*., 2009; van Erp *et al*., 2014; Hills, 2004). In glycolysis pathway, G6P is then converted to pyruvate, which is subsequently imported into the chloroplast to synthesize acetyl‐CoA, a precursor in fatty acid synthesis, via the activity of pyruvate dehydrogenase complex (Durrett *et al*., 2008; Vigeolas *et al*., 2007). The newly synthesized acetyl‐CoAs are then converted to malonyl‐CoA via the activity of acetyl‐CoA carboxylase (ACCase‐α) and then the fatty acid synthase enzymes, namely 3‐ketoacyl Acyl carrier proteins (ACP) are involved to condensate acetyl‐CoA and malonyl‐ACP. Subsequently, the fatty acyl chains are released from ACP via hydrolysis catalysed by acyl‐ACP thioesterases. The synthesized fatty acyl‐CoAs exported from the plastids are utilized in the stepwise esterification of the glycerol backbone via the Kennedy pathway to synthesize glycerolipids, including TAGs at the endoplasmic reticulum (Durrett *et al*., 2008; Kennedy, 1961). TAG assembly is initiated by acylation of glycerol‐3‐phosphate (Gly3P) by Gly3P acyltransferase (GPAT) to lysophosphatidic acid, which is subsequently acylated to phosphatidic acid (PA) by lysophosphatidic acyltransferase (LPAT). PA is then dephosphorylated to form diacylglycerol (DAG) by phosphatidic acid phosphohydrolase (PAH). DAG acyltransferases (DGAT) finally esterify DAG to produce TAG, which is ultimately stored in ER‐derived oil bodies (Lacey *et al*., 1998; Vigeolas *et al*., 2007).

In developing seeds of oilseed crops, DGAT catalyses the acylation of the sn‐1,2‐diacylglycerol (DAG) to form TAG, which is the final committed step in the Kennedy pathway (Kennedy, 1961). Biochemical analysis and transgenic studies of lipid metabolism in several oilseed crops have evidenced that DGAT activity has a considerable effect on carbon flow into seed oil and it appears to be crucial for mediating seed oil biosynthesis in quantitative and qualitative manners (Taylor *et al*., 2009; Weselake *et al*., 2008). Compared with other enzymes in lipid biosynthesis, DGAT exhibited relatively low activity, and the accumulation of its substrate DAG in developing seeds suggests that DAG‐to‐TAG acylation reaction represents a rate‐limiting step in seed oil formation (Perry *et al*., 1999; Taylor *et al*., 2009). Accordingly, cloning of genes encoding distinct oilseed DGATs has been reported, and seed‐specific overexpression of DGATs has resulted in an increase in DGAT activity and subsequently seed oil content (Aznar‐Moreno *et al*., 2015; van Erp *et al*., 2014; Jako *et al*., 2001; Kim *et al*., 2016; Taylor *et al*., 2009).

DGAT1 enzyme was evidenced to be a major determining factor for oil quantity and fatty acid composition of seed oils in several crops, and manipulation of its gene expression has proven to be one of the successful approaches utilized to increase oil content and alter the fatty composition (Jako *et al*., 2001; Kim *et al*., 2016; Taylor *et al*., 2009; Xu *et al*., 2008). However, structural and functional motif analysis of DGAT1 from *Tropaeolum majus L*. and Arabidopsis also revealed multiple potential motifs as target sites of members of the sucrose nonfermenting (SNF)‐related protein kinase 1 (SnRK1) family, suggesting the regulation of DGAT1 activity by post‐translational modification (i.e. phosphorylation), which may down‐regulate DGAT1 activity and affect seed oil production (Xu *et al*., 2008; Zou *et al*., 1999). It is suggested to enhance DGAT1 activity by blocking the SnRK1 target site via site‐directed mutagenesis (SDM), thus preventing phosphorylation. Accordingly, the substitution of Ser^197^ to Ala of SnRK1 site in *T. majus* DGAT1 has increased its activity by 38%–80%, and when the modified *DGAT1* gene was expressed into Arabidopsis seeds, it enhanced the seed oil production in transgenic plants by 3%–10%, seed weight by 45% and total oil yield by 51% on dry weight basis (Xu *et al*., 2008). This crucial impact of SDM on DGAT1 activity would encourage researchers to undertake similar approach to up‐regulate DGAT1 activity by targeting a putative SnRK1 site, thus enhancing DGAT1 impact in seed oil production. However, the negative regulation of DGAT1 activity via phosphorylation of Ser via SnRK1 has not been confirmed yet *in planta*, the point which requires further investigations.

Utilizing genetic and biochemical knowledge of lipid metabolism, several studies have established the possibility to increase seed oil contents and/or alter oil composition by manipulating the expression levels of individual enzymes involved in oil biosynthesis (Dalal *et al*., 2015; van Erp *et al*., 2014; Jako *et al*., 2001; Kelly *et al*., 2013; Li *et al*., 2015; Taylor *et al*., 2009; Vigeolas *et al*., 2007; Zou *et al*., 1997). Nevertheless, the multiple steps involved in the flow of carbon flux through oil metabolic pathways and the sensitivity of the seed oil content to multiple metabolic reactions render the impact of this approach theoretically limited (van Erp *et al*., 2014; Tang *et al*., 2012; Weselake *et al*., 2008). Over the years, several attempts have been made to significantly boost the seed oil content in various oilseed crops via biotechnological approaches, and many of these attempts concentrated on increasing fatty acids production/use rates or TAG assembly. For instance, manipulating a key rate‐limiting enzyme involved in fatty acid biosynthesis, ACCase‐α resulted in 5% increase in seed oil in oilseed rape (*Brassica napus* L., Roesler *et al*., 1997). Further improvement in seed oil content has been reported through manipulating TAG synthesis pathways. Overexpressing a yeast *sn*‐2 acyltransferase gene (namely, LPAT) has resulted in 8%–48% and 3.2% increases in seed oil content in oilseed rape and soybean (*Glycine max* (L.) Merr., Zou *et al*., 1997; Taylor *et al*., 2002; Rao and Hildebrand, 2009), respectively. Further, targeting a diacylglycerol acyltransferase (DGAT1), seed oil content has substantially increased as reported in several transgenic crops, including Arabidopsis (~30%, Jako *et al*., 2001), oilseed rape (~3–8%, Taylor *et al*., 2009; Xu *et al*., 2008) and Camelina (~24%, Kim *et al*., 2016) as compared to nontransgenic plants, on a dry weight basis. Furthermore, a successful increase in overall TAG accumulation was accomplished mostly by targeting the *WRINKLED 1* (*WRI1*), a master transcriptional regulator of genes involved in FA synthesis and glycolysis (10%–20% increase compared to WT plants (van Erp *et al*., 2014).

Several oilseed crops have been investigated concerning oil metabolism and were targeted for improved seed and oil qualities. Among those crops, *Camelina sativa* (L.) Crantz, an oilseed crop, which belongs to Brassicaceae family, has attracted increasing interests in the last decades due to its positive agronomic attributes, geographic suitability, genetic engineering feasibility, availability of genetic information and the wide range of applications for its oil and its bioproducts (Putnam *et al*., 1993; Séguin‐Swartz *et al*., 2009; Li *et al*., 2015; Abdullah *et al*., 2016; Kang *et al*., 2011; Lu and Kang, 2008; Kagale *et al*., 2014; Nguyen *et al*., 2013). Unlike the case in other oilseed crops, few reports on enhancement of seed yield and seed oil content via genetic engineering of *C. sativa* have been demonstrated (An and Suh, 2015; Dalal *et al*., 2015; Kim *et al*., 2016; Li *et al*., 2015; Zhang *et al*., 2012). These reports have mainly concentrated on genes involved in reducing photorespiration and modulating carbon metabolism, which indirectly impacts seed oil content (Dalal *et al*., 2015; Zhang *et al*., 2012), a gene encodes the Arabidopsis *WRl1* (Kim *et al*., 2016) and a gene encodes a patatin‐related phospholipase AIIIδ (*pPLAIII*δ), which involved in phosphatidylcholine turnover (Li *et al*., 2015).

In contrast to the several reports on *DGAT1* and other TAG‐related genes, only a few reports have studied the critical role of Gly3P, the second substrate needed for TAG synthesis. It was reported in developing seeds of Arabidopsis and oilseed rape that the rate of Gly3P supply is not sufficiently rapid to maintain high Gly3P levels during fast oil accumulation stage in seeds (Gibon *et al*., 2002; Vigeolas and Geigenberger, 2004). Crucially, increasing Gly3P levels in developing rape seeds fed with glycerol has resulted in an increase in the carbon flux to TAG, suggesting that Gly3P appears to co‐limit the rate of TAG formation in seeds (Vigeolas and Geigenberger, 2004). Overexpression of a cytosolic *glycerol‐3‐phosphate dehydrogenase* (*Gly3PDH*) gene from Yeast (*Saccharomyces cerevisiae*) into oilseed rape has significantly increased Gly3P activity, resulting in up to 40% increase in seed fatty acid content (Vigeolas *et al*., 2007).

Therefore, to increase seed oil content in Camelina, we used a transgenic approach to investigate the importance of Gly3P supply for use as the backbone for TAG synthesis, and the importance of acylation of fatty acids in the downstream process for TAG synthesis. For this purpose, we overexpressed a yeast gene coding for cytosolic Gly3PDH (GPD1) under the control of the seed‐specific *oleosin* promoter, and the Arabidopsis *DGAT1* gene, either in its native or modified form, to draw fatty acids into TAG, under the control of seed‐specific *glycinin* promoter, in transgenic Camelina. Further, we investigated the effect of stacking these two genes on achieving a synergistic effect on the flux through the TAG synthesis pathway, and thereby further increase the oil yield.

## Results

### Overexpression of ScGPD1 and AtDGAT1 into *C. cativa*

**Figure 1:**
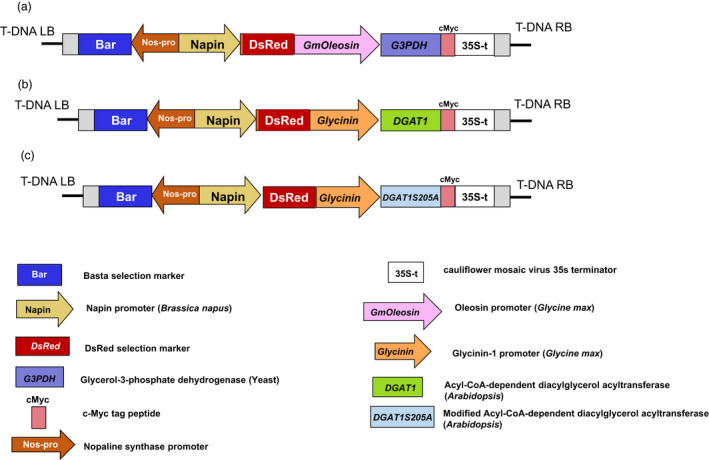
T‐DNA insertions used to transform *Camelina sativa*. Shown here are the pBnRGW RedSeed binary vectors containing seed‐specific cassettes for expression of the *glycerol‐3‐phosphate dehydrogenase*, *GPD1* (a) and the *diacylglycerol acyltransferase*, *DGAT1* and *DGAT1m* (b and c). DsRed fluorescence marker and the herbicide‐resistant *bar* gene (Basta containing phosphinothricin) for selection of transformants are shown in the constructs.

For the metabolic engineering of Camelina seeds for increased levels of triacylglycerols, *GPD1* and *DGAT1* genes were introduced into Camelina nuclear genome under the control of *oleosin* and *glycinin* promoters from soybean, respectively. Further, to test whether the mutation in DGAT1, where serine^205^ was substituted with alanine, can enhance its enzymatic activity and positively impact seed oil content, the modified *DGAT1S205A* (referred here as *DGAT1m*) was also introduced into Camelina under soybean *glycinin* promoter, following similar approach reported in Xu *et al*. (2008). Furthermore, to test whether combined expression of *GPD1* and *DGAT1* or *DGAT1m* can further increase the oil yield, Camelina plants were co‐transformed with *DGAT1* or *DGAT1m* and *GPD1* constructs (referred here as GPD1 + DGAT1 and GPD1 + DGAT1m, Figure 1).

**Figure 2:**
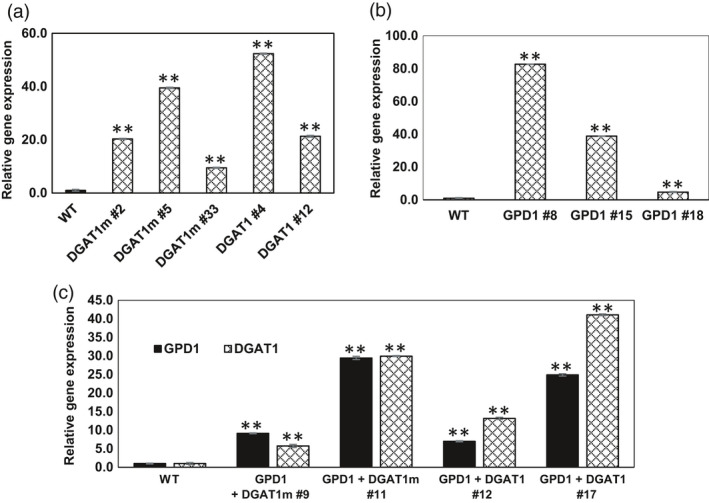
Analysis of *DGAT1* and *GPD1* transcript expression in Camelina developing seeds of T3 generation in the transgenic lines overexpressing AtDGAT1 (a), ScGPD1 (b) and DGAT1 + GPD1 (c). Values are the mean ± standard error (*n* = 3). Asterisks denote significance of differences between WT and transgenic lines (Student's *t*‐test): **P < 0.01; *P < 0.05.

The expression of transgenes in Camelina plants was verified by quantitative real‐time qRT‐PCR. T1 Transgenic plants showing DsRed fluorescence were sown into the soil to obtain future generations, and DsRed fluorescence was used to identify homozygous lines. T1 plants were also tested for the presence of *oleosin* and *glycinin* promoters in GPD1 and DGAT1 overexpressors, respectively, by PCR analysis (Figure S1A, B and C). Developing seeds (16‐21 DAF) of T3 homozygous lines were used to extract RNA for confirming the expression of *DGAT1* and *GDP1* transcripts using RT‐PCR. The relative expression of *DGAT1*,* DGAT1m* and *GPD1* transcripts was 10‐ to 40‐fold higher in Camelina transgenic lines than in wild‐type (WT) controls (Figure 2a and b). Similarly, the expression levels of *DGAT1*,* DGAT1m* and *GPD1* in GDP1 + DGAT1 and GDP1 + DGAT1m lines were ~10‐fold higher than WT controls (Figure 2c). These results confirmed that *DGAT1*,* DGAT1m* and *GPD1* genes were successfully integrated and expressed at higher levels in transgenic lines.

### Seed‐specific overexpression of AtDGAT1 and ScGPD1 increased seed mass, seed size and seed yield in transgenic *C. sativa*

**Figure 3:**
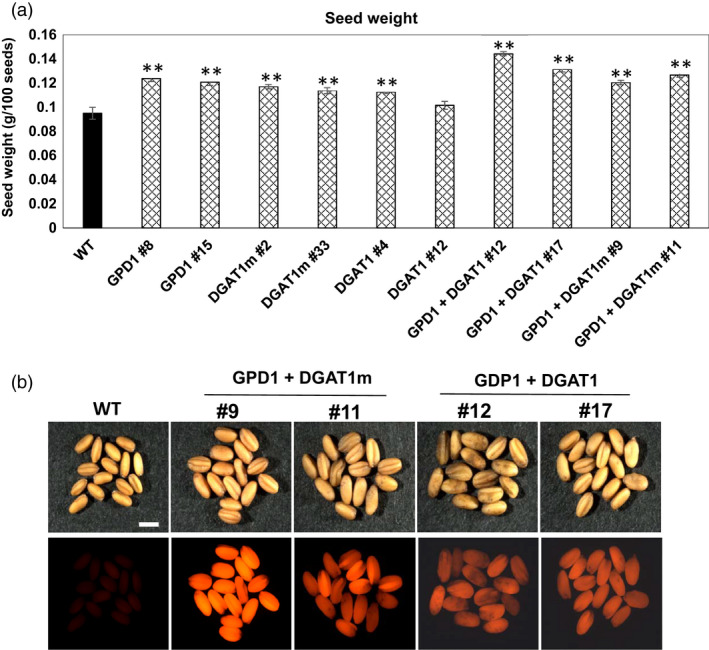
Individual and combined effects of GPD1 and DGAT1 expression on average seed mass of homozygous T3 seeds in transgenic Camelina plants. (a) The seed weight (g/plant) of Camelina lines overexpressing AtDGAT1, ScGPD1 and WT controls. (b) Images of transgenic and WT seeds illuminated under DsRed fluorescence filter. Values in (a) are means ± SE on measurements on seeds from individual plants (*n* = 4–10) of each genotype grown in controlled conditions. Bar = 1 mm. Asterisks denote significance of differences between WT and transgenic lines (Student's *t*‐test): **P < 0.01; *P < 0.05.

To examine whether overexpression of DGAT1 and GPD1 can cause morphologic changes in Camelina seeds, the size and weight of transgenic and WT seeds were measured. Analysis of 100‐seed weight indicated that overexpression of GPD1, DGAT1 or DGAT1 m has altered seed morphology regarding significant increases (*P* < 0.05) in seed mass (Figure 3). Transgenic lines expressing GDP1, DGAT1 or DGAT1m, individually, showed 19%–30% increase in seed mass. The maximum increase in seed mass was observed in Camelina lines co‐expressing GPD1 + DGAT1 (up to 52%) or GPD1 + DGAT1m (up to 33%), relative to the WT seeds (Figure 3a). This increase in seed weight in the co‐transformed lines appears to be associated with a significant increase in their seed sizes as compared to WT seeds (Figure 3b).

**Figure 4:**
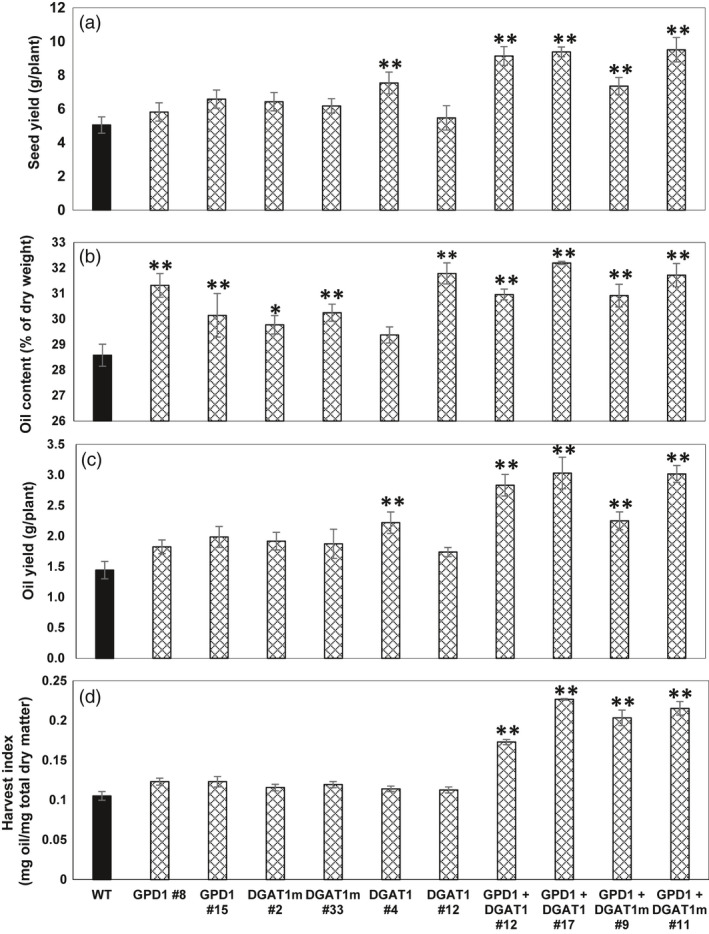
Individual and combined effects of GPD1 and DGAT1 expression on average seed yields (a), % oil content (b), oil yield (c) and oil harvest index (d) of T3 homozygous seeds in transgenic Camelina plants. Values are means ± SE on measurements on seeds from individual plants (*n* = 4–10) of each genotype grown in controlled conditions. Asterisks denote significance of differences between WT and transgenic lines (Student's *t*‐test): **P < 0.01; *P < 0.05.

Total per plant seed yield of T3 homozygous transgenic lines was measured relative to WT seeds (Figure 4a). Unlike the significant increases in seed mass seen in almost all of the transgenic lines overexpressing GPD1, DGAT1 or DGAT1m, individually, no significant difference in seed yield obtained in those lines, except the transgenic line DGAT1 #4, which attained 49% more seed yield as compared to WT, whereas the cotransformed GDP1 + DGAT1 and GPD1 + DGAT1m lines showed the maximum gain in per plant seed yield compared to WT plants. Both GPD1 + DGAT1 and GPD1 + DGAT1m lines exhibited 86%–88% gain in seed yield under glasshouse conditions (Figure 4a).

### Seed‐specific overexpression of ScGPD1 and AtDGAT1 increases seed oil content but causes no change in seed protein content

Seeds of T3 homozygous Camelina lines transformed individually with GPD1, DGAT1 and DGAT1m or co‐transformed (GPD1 + DGAT1 and GPD1 + DGAT1m) were analysed for seed percentage oil content using Minispec mq‐20 20 MHz NMR spectroscopy to quantify total seed oil content (wt./wt., Figure 4b). All Camelina lines co‐expressing DGAT1 and GPD1 exhibited statistically significant (*P* < 0.05) increases in oil content per dry seed weight compared to the WT controls. On average, seed oil content in those lines was approximately 8%–13% higher compared to that in WT (Figure 4b), and some individual lines exhibited up to 20% increase in seed oil content (seed oil content was 34.5% in transgenics vs. 28.5% in WT; line GPD1 + DGAT1 #12‐3‐11; see Table S1). Further, seed oil content in Camelina lines overexpressing the modified DGAT1 was also increased by ~ 8.5% compared to an increase of 5.6% in DGAT1 lines and an increase of 5.4% in GPD1 lines, as compared to WT plants, on average basis (Figure 4b and Table S1). Further, seeds of some of the individual transgenic lines overexpressing either GDP1, DGAT1 or modified DGAT1m also led up to an average 11% increase in oil contents as compared to WT (Figure 4b and Table S1).

Due to the finding that the seed weight of Camelina is more than 40% of the per plant total above‐ground biomass, expression of percentage increase in seed oil yield as a percentage of total above‐ground dry biomass severely underestimates the total oil yield. Therefore, we expressed the per plant total oil yield based on the % oil contents and total seed weight for all T3 homozygous transgenic Camelina lines. Determining the total seed yield in grams per plant (Figure 4a) helped us to derive total oil yield (grams per plant), and by multiplying the total seed oil content by the total seed yield per plant, it was possible to determine the total oil yield per plant basis (Figure 4c and Table S1). The results indicated that Camelina transgenic lines, which exhibited significant increases in total seed yield per plant (Figure 4a), also displayed significant increases (*P* < 0.05) in total oil yield per plant as compared to the WT plants.

Interestingly, the highest oil yield was obtained in seeds of Camelina transgenic lines co‐expressing GPD1 and DGAT1. The per plant oil yield was approximately doubled in GPD1 + DGAT1 and GPD1 + DGAT1m lines than that in WT (up to 3.0 g in transgenic lines vs. 1.44 g in WT, Figure 4c and Table S1). However, overexpressing DGAT1 or GPD1 alone, except line DGAT1 #4, observed slight, but not significant, increases in total oil yield per plant in GPD1, DGAT1, DGAT1m transgenics despite the increased percentage of seed oil content seen in their seeds compared to WT seeds. Our results suggest that an increase in seed oil content and seed yield in Camelina can lead to an overall increase in Camelina oil yield under glasshouse conditions.

Furthermore, the oil harvest index, which is used in agriculture to quantify the oil yield versus the total plant biomass (Vafaei *et al*., 2010), was determined in Camelina transgenic lines in comparison with WT plants (Figure 4d). The oil harvest index exhibited no changes in Camelina transgenic lines overexpressing GPD1, DGAT1 or DGAT1m alone compared to their relative WT. However, as expected, transgenic lines GPD1 + DGAT1 and GPD1 + DGAT1m exhibited significantly higher oil harvest index (up to twofold, *P* < 0.05) in both GPD1 + DGAT1 and GPD1 + mDGAT1 lines than that in WT. Altogether, these findings indicate that the seed‐specific co‐expression of GPD1 and DGAT1 enhanced the overall seed oil yield in Camelina plants.

**Figure 5:**
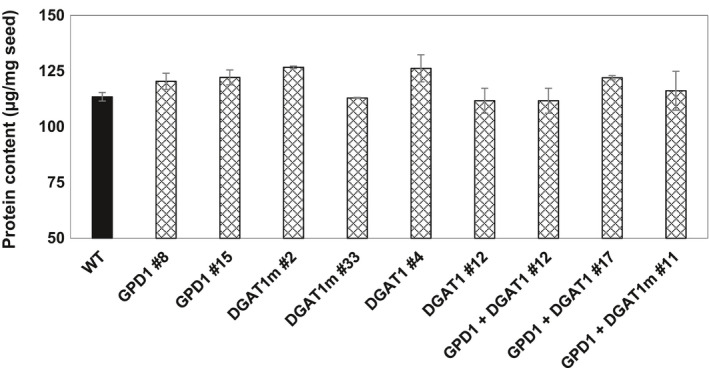
Individual and combined effects of GPD1 and DGAT1 expression on protein content in Camelina seeds. Values are means ± *SE* on measurements on seeds from individual plants (*n* = 4) of each genotype grown under controlled conditions.

Moreover, to investigate the influence of increased seed oil content in Camelina transgenic lines on the amount of seed storage proteins, transgenic lines and WT seeds were further analysed for protein content (Figure 5). The results indicated that the expression of GPD1 and DGAT1, either individually or in a combination, has led to no significant changes in the protein content in Camelina mature seeds.

### Overexpression of GDP1 and DGAT1 altered fatty acid composition

**Figure 6:**
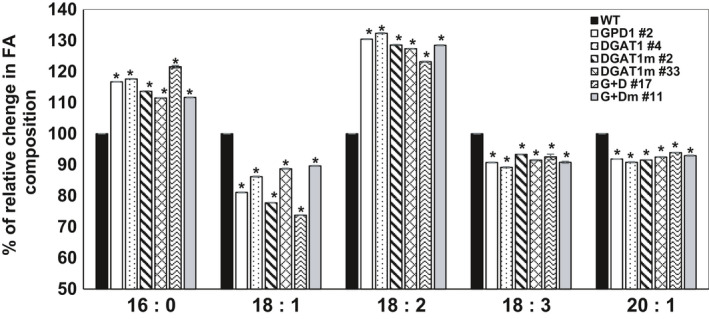
Relative change in FA composition in dried transgenic and WT Camelina seeds analysed by gas chromatography. The percentage relative increase or decrease in the levels of palmitic acid (C16:0), oleic acid (C18:1), linoleic acid (C18:2), linolenic acid (C18:3) and eicosenoic acid (C20:1) in transgenic Camelina lines overexpressing AtDGAT1 and ScGPD1, individually or in a combination, as compared to nontransgenic WT are shown. Values are means ± SE (*n* = 4). WT values are normalized to the threshold 100. Asterisks denote significance of differences between WT and transgenic lines (Student's *t*‐test): **P < 0.01; *P < 0.05.

An increased expression of GPD1 and DGAT1 could affect the type of fatty acids (FAs) incorporated into the glycerol backbone via the activity of acylglycerol acyltransferase enzymes, that is GPATs, LPATs and DGATs. Therefore, to evaluate the effect of GPD1 and DGAT1 overexpression in Camelina, a FAME analysis was applied on mature seeds of transgenic and WT plants, and both the content and composition of FAs were determined (Figure 6 and Table 1). Fatty acid composition of the seed oil in WT plants revealed an average content of palmitic (C16:0, 7%), stearic (C18:0, 2.7%), oleic (C18:1, 14%), linoleic (C18:2, 16.5%), α‐linolenic (C18:3, 40%) and gondoic (C20:1, 11.5%) acids, which is in agreement with the FA profile previously reported in Camelina oil (Abdullah *et al*., 2016; Kim *et al*., 2016; Li *et al*., 2015; Rodríguez‐Rodríguez *et al*., 2013).

**Table 1:** Fatty acid composition and content of Camelina oil of transgenic and wild‐type plants

Fatty acid		WT	GPD1 #2	DGAT1 #4	DGAT1 m #2	DGAT1m #33	GPD1 + DGAT1 #17	GPD1 + DGAT1m #11
16:0	%	7.19 ± 0.02	**8.39 **±** **0.05	**8.46 **±** **0.10	**8.17 **±** **0.15	**8.02 **±** **0.07	**8.74 **±** **0.41	**8.03 **±** **0.10
	nmol	99.14	118.36	128.09	119.27	126.03	104.35	113.87
18:0	%	2.79 ± 0.04	3.14 ± 0.03	3.12 ± 0.11	2.88 ± 0.07	2.94 ± 0.09	2.89 ± 0.13	2.86 ± 0.06
	nmol	38.51	44.18	47.14	42.40	46.30	34.53	40.59
18:1	%	14.07 ± 0.01	**11.41 **± 0.12	**12.12 **±** **0.12	**10.94 **±** **0.06	**12.48 **±** **0.13	**10.38 **±** **0.08	**12.61 **±** **0.13
	nmol	193.88	160.83	184.09	160.18	195.95	126.47	179.75
18:2	%	16.57 ± 0.02	**21.62 **±** **0.12	**21.94 **±** **0.16	**21.31 **±** **0.18	**21.11 **±** **0.07	**20.41 **±** **0.18	**21.29 **±** **0.14
	nmol	228.43	304.76	332.27	312.54	331.96	250.40	302.62
18:3	%	39.67 ± 0.07	**36.00 **±** **0.14	**35.38 **±** **0.18	**37.03 **±** **0.09	**36.30 **±** **0.18	**36.72 **±** **0.85	**36.03 **±** **0.31
	nmol	546.86	507.65	536.50	541.88	570.72	453.89	512.78
20:0	%	1.51 ± 0.01	**1.87 **±** **0.04	**1.82 **±** **0.03	**1.72 **±** **0.05	1.67 ± 0.05	**1.78 **±** **0.05	1.64 ± 0.05
	nmol	20.87	26.37	27.60	25.38	26.37	21.65	23.35
20:1	%	11.53 ± 0.03	**10.59 **±** **0.01	**10.47 **±** **0.15	**10.56 **±** **0.05	**10.67 **±** **0.07	**10.83 **±** **0.09	**10.72 **±** **0.15
	nmol	158.93	149.35	158.99	154.71	167.97	131.96	152.96
20:2	%	1.34 ± 0.0	**1.91 **± 0.01	1.79 ± 0.02	**1.98 **±** **0.01	1.83 ± 0.01	**2.01 **±** **0.02	1.53 ± 0.34
	nmol	18.45	26.96	27.19	28.95	28.87	24.47	22.24
20:4	%	2.64 ± 0.03	**2.35 **±** **0.02	**2.26 **±** **0.03	2.60 ± 0.02	**2.31 **±** **0.04	**2.78 **±** **0.04	**2.29 **±** **0.04
	nmol	36.31	33.10	34.29	38.03	36.51	33.80	32.68
24:1	%	0.52 ± 0.01	0.50 ± 0.01	0.51 ± 0.01	0.54 ± 0.01	0.50 ± 0.01	0.67 ± 0.08	0.50 ± 0.02
	nmol	7.10	7.10	7.76	7.85	7.84	7.69	7.04
Others	%	2.18	2.22	2.12	2.28	2.17	**2.78**	2.49
	nmol	30.08	31.33	32.10	33.25	34.18	32.80	34.89

Data represent the mean of the three independent measurements ± standard errors, in nmol per dry weight and in percentage %. C16:0 palmitic acid; C18:0 stearic acid; C18:1 oleic acid; C18:2 linoleic acid; C18:3 α‐linolenic acid; C20:0 arachidonic acid; C20:1 gondoic acid; C20:2 eicosadienoic acid; C20:4 arachidonic acid; C24:1 nervonic acid. Others account for C14:0 myristic acid; C16:1 palmitoleic acid; C22:0; C20:3 mead acid; C22:2 docosadienoic acid; C20:5 eicosapentaenoic acid; and C24:0 tetracosanoic acid. The significance of the effect of the genotypes on FA profiles was tested statistically by Dunnett's test at *P* < 0.05 compared with the WT, and comparisons significant at the 0.05 level are highlighted in bold.

Nevertheless, the expression of GPD1 and DGAT1 revealed significant changes in FA concentrations and their saturation levels, with similar patterns of changes observed in all examined transgenics (Figure 6 and Table 1). Expression of GPD1 resulted in significant increases in the levels of C16:0 and C18:2 acids (up to 17% and 31%, respectively), and this incline was associated with significant decreases in the levels of C18:1, C18:3 and C20:1 acids (up to 19%, 9% and 8%, respectively) in GPD1 lines as compared to WT. Similarly, the FA profile in DGAT1 lines showed an increased levels of C16:0 and C18:2 acids (up to 18% and 33%, respectively), and decreased levels of C18:1, C18:3 and C20:1 acids (up to 14%, 11% and 11%, respectively) compared to that in WT. Co‐expression of GPD1 and DGAT1 also showed the same pattern of changes with even a further decrease in the level of C18:1 acid (up to 36%) as compared to WT.

### GDP1 and DGAT1 overexpression has no effect on seed germination and early seedling growth

**Figure 7:**
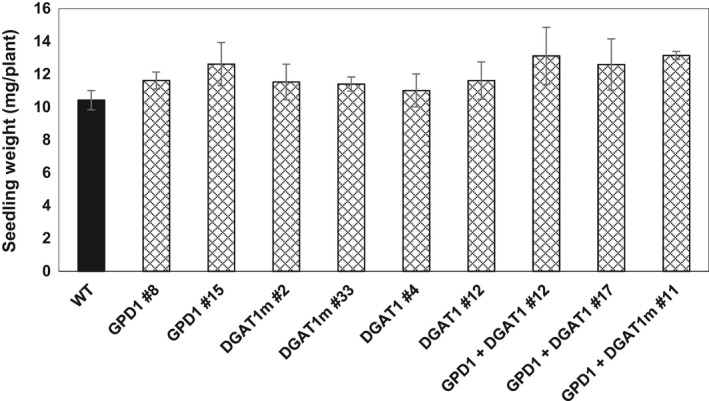
Early seedling growth rate of Camelina in the T3 transgenic and WT Plants. Values are means ± S.E. of measurements on seeds from individual plants (*n* = 12) of each genotype grown under controlled condition over germination papers.

To determine whether the seed‐specific overexpression of GPD1 and/or DGAT1 was associated with any negative or positive impacts on seed vigour, an important agronomic trait, seeds of transgenic lines and WT plants were germinated. The average time for 50% of seeds to germinate was recorded, and then the growth of 5‐day‐old seedlings was measured as mg per plant (Figure 7). Results indicated that it takes approximately 12 h for 50% of the seeds to germinate, and all the seeds were germinated after 18 h, with no difference in germination rates between transgenic and WT seeds (Figure 7 and Figure S2). The initial rates of seedlings growth, under the growth conditions applied herein, also showed no significant differences in seedlings fresh weight between transgenic and WT plants (Figure 7). These findings suggest that overexpression of DGAT1 and/or GPD1 has no detrimental effects to seed vigour under laboratory conditions.

## Discussion

The present study shows that the seed‐specific overexpression of the cytosolic *GPD1 gene* from *S. cerevisiae*, to provide Gly3P for TAG assembly, and *A. thaliana DGAT1*, which catalyses the final step in the TAG assembly from DAG and acyl‐CoA, has positive effects on seed oil content, seed weight, and seed yield in *C. sativa*. Transgenic *C. sativa* plants overexpressing *DGAT1 or DGAT1m* gene under the control of *glycinin* promoter have observed averages of ~23% increase in seed mass and ~6%–11% increase in seed oil contents, whereas overexpression of *GPD1* under the control of *oleosin* promoter has resulted in significant increases in seed mass (up to 30%) and seed oil contents (up to 10%). Co‐expression of these two genes in Camelina further enhanced seed mass (up to ~52%), seed oil content (up to ~13%) and total seed yield per plant (up to ~46%–88%), as compared to the nontransformed WT plants. These significant increases in seed oil content and seed yield can be translated into a greater oil yield and oil harvest index, on a dry matter basis, although the relationship seems to be less than proportionate and is limited by the experimental conditions applied in this study. Our findings are in agreement with the previous reports, which investigated the positive effects on seed and oil yields in other oilseed crops via engineering multiple steps in the TAG metabolic pathway. Overexpression of *A. thaliana DGAT1* under the control of seed‐specific promoter has been reported to boost seed oil content in Arabidopsis and Canola (van Erp *et al*., 2014; Jako *et al*., 2001; Taylor *et al*., 2009). Further, DGAT1 from other plant species, when overexpressed, has also increased the storage oil content in transgenic oilseed rape (*Brassica napus L*., Weselake *et al*., 2008), maize (*Zea mays* L., Zheng *et al*., 2008), garden nasturtium (*Tropaeolum majus* L., Taylor *et al*., 2009) and Camelina (Kim *et al*., 2016).

By contrast, to our knowledge, only a unique study has investigated the overexpression of yeast cytosolic GPD1 in transgenic *B. napus* under the control of the seed‐specific *napin* promoter, despite the importance of Gly3P supply as the second substrate essential for TAG assembly (Vigeolas *et al*., 2007). Furthermore, few studies have investigated the importance of stacking TAG pathway‐related genes in order to enhance seed oil content in oilseed crops. Among those, a study by van Erp and his colleagues has showed that stacking Arabidopsis *DGAT1* and *WRl1* genes, combined with suppression of the TAG lipase, *SUGAR‐DEPENDENT1* (*SDP1*) gene, observed a higher percentage seed oil content and a higher seed mass in transgenic Arabidopsis compared to WT plants (van Erp *et al*., 2014). Consistent with their findings, co‐expression of *GPD1* and *DGAT1* genes herein has resulted in comparable further increases in seed oil content, seed mass and seed yield in Camelina transgenic lines.

Increasing the expression of cytosolic *GPD1* in transgenic Camelina under the control of *oleosin* promoter has significantly altered the seed mass in all the lines tested, with three lines exhibited considerable increases in seed oil content (Figures 3 and 4). It was reported that GPD1*,* when overexpressed in oilseed rape, has led to a twofold increase in Gly3PDH activity, which significantly increases the supply of Gly3P as a precursor for TAG assembly (Vigeolas *et al*., 2007). This increase in Gly3P supply is correlated with the increase in FA content in mature seeds and the decrease in Gly3PDH's potential substrate dihydroxyacetone phosphate (DHAP) by the direct conversion of DHAP into Gly3P catalysed by Gly3PDH activity (Vigeolas *et al*., 2007). We believe that a possible interpretation of increasing seed mass and stimulating TAG synthesis in Camelina seeds overexpressing GPD1 could be the increase in embryo weight, which is not measured herein, due to the increased lipid content and the continuous supply of Gly3P for TAG assembly.

Similarly, increasing seed oil content in Camelina lines overexpressing DGAT1 could be as a result of the correspondent increase in DGAT1 enzyme activity as previously discussed (Kim *et al*., 2016; Taylor *et al*., 2009). As DGAT1 catalyses the conversion of DAG and acyl‐CoA into TAG in the ER, the increased levels of DGAT1 activity, while its substrate DAG is redundant, can extensively increase the amount of TAGs accumulated in mature seeds. However, the high expression of *DGAT1* gene can also have an adverse effect, as it also affects the substrate–enzyme–product relationship, thus causing a limitation in certain substrates in TAG metabolic pathway than others (Taylor *et al*., 2009). Combining the expression of both GPD1 and DGAT1 in Camelina seeds stimulates oil synthesis, possibly through the increased supply of Gly3P, which can directly provide the required glycerol backbone for TAG assembly, and indirectly enrich the amount of acyl‐CoA pools (Vigeolas *et al*., 2007). Therefore, G3P and acyl‐CoAs would become more available to the downstream enzymes in TAG metabolic pathways, including DGAT1, which incorporate the acyl‐CoAs into the final TAGs. This stimulation of oil synthesis appears to require the up‐regulation of the enzymes involved in controlling the committed steps in TAG assembly. Relatively, in agreement with the previous report by Sharma *et al*. (2008), overexpression of Arabidopsis DGAT1 in *B. napus* was associated with increasing the transcription of several genes involved in the Kennedy pathway for TAG synthesis, including *GPAT1*,* LPAT*,* PAH* and *DGAT*1. Moreover, RNA‐Seq analysis of Camelina transcriptome during seed development has indicated higher expressions for a GPAT family member (*GPAT9*), PP family members (*PAH1* and *PAH2*) and *DGAT1*, but not *LPATs* (Abdullah *et al*., 2016). It could be the case here that these active enzymes are responsible for the committed flow of metabolites, by acting as sinks, towards increased levels of TAG in Camelina seeds overexpressing GPD1 and DGAT1. Furthermore, the increased seed weight and size in Camelina transgenics could be as a result of the growth of cotyledonary embryonic cells due to the accumulation of TAG molecules as previously reported (Kim *et al*., 2016).

Site‐directed mutagenesis (SDM) has been applied in previous reports as an efficient tool to modify putative functional motifs in DGAT1 enzyme to up‐ or down‐regulate its activity causing effective changes in seed and oil qualities (Katavic *et al*., 1995; Xu *et al*., 2008; Zou *et al*., 1999). As mentioned earlier in the introduction section, in an attempt to increase *T. majus* DGAT1 activity, Xu and his coworkers substituted Ser^197^ with Ala at the putative SnRK1 serine/threonine protein kinase target site for dephosphorylation. Overexpression of the mutated TmDGAT1 in Arabidopsis seeds resulted in ~38%–80% increase in DGAT1 activity and 3%–10% higher seed oil content in the transgenic seeds on a seed DW basis (Xu *et al*., 2008). Following similar approach, in the current research, we modified DGAT1 in the putative SnRK1 target site, and the Ser^205^ to Ala mutant caused a slight, but not significant, increase in the levels of seed oil content in the modified DGAT1m lines compared to that in the native DGAT1 lines. Unlike Xu *et al*. (2008) findings, we did not find any significant difference in seed yield, seed size or overall plant growth between DGAT1 and DGAT1m lines. The inconsistency between the impact of DGAT1 modification detected herein and the previous report by (Xu *et al*., 2008) could be due to various factors, including the different DGAT1 enzyme (*A. thaliana* vs. *T. majus*) used for mutagenesis in our study, the putative functional motif(s) targeted for phosphorylation, the structural integrity surrounding this motif as well as the factors that control the enzymatic reactions, such as the availability and concentration of DGAT enzyme substrates, pH, temperature (Guo *et al*., 2001; Tang *et al*., 2005; Xu *et al*., 2008). Additionally, our analysis of the amino acid sequences of Arabidopsis DGAT1 (At3g54320) revealed several putative phosphorylation target motifs via kinases (data not shown). As we targeted only one serine at position 205 in the current study, there may be other sites as targets for kinases that could alter the activity of AtDGAT1. Further studies are needed to better understand the potential impact of post‐translational modifications on AtDGAT1 enzyme activity and how it can be translated into effective improvement in seed and oil qualities.

In oilseed crops, the fatty acid composition of oils is determined by the substrate preference of DGATs and the availability of the acyl‐CoA species in the ER lumen (Kim *et al*., 2016). Analysis of seed FAs in Camelina WT plants revealed FAME contents and composition similar to the previous reports (Rodríguez‐Rodríguez *et al*., 2013; Kim *et al*., 2016; Li *et al*., 2015). The FAME analysis of the selected GPD1‐ and DGAT1‐overexpressing lines has indicated significant increases in C16:0 and 18:2 levels and significant decreases in C18:1, C18:3 and C20:1 levels as compared to nontransformed WT. This FAME profile is in contrast to the FAME profile reported in Camelina transgenics overexpressing CsDGAT1B, which accumulated higher C18:1 and C18:3 while the level of C18:2 was lower as compared to nontransgenic plants (Kim *et al*., 2016). This disagreement could be due to the varied redundancy of acyl‐CoA species and/or substrate preferences for Arabidopsis DGAT1 used in this study, compared to the indigenous Camelina CsDGAT1B used in Kim *et al*. (2016). Conversely, unlike the previous reports on the impact of DGAT1 overexpression on FA profile, there are no data of altering GPD1 expression in Camelina to compare with; however, the yeast GPD1 overexpression did not change the fatty acid composition of the seed oil in transgenic oilseed rape (Vigeolas *et al*., 2007). Further, the observed increase in the levels of linoleic acid (up to 33%) and the decrease in α‐linolenic acid (up to 11%) in seed oils of Camelina transgenics would further improve the effectiveness of Camelina oil as a healthier ingredient in food and feedstocks as well as for a relatively better biodiesel blend (Rodríguez‐Rodríguez *et al*., 2013).

The oil content of Camelina mature seeds ranges from 30% to 40% of the seed weight, and this wide range appears to be dependent on several factors, including genetic background, geographic and climatic conditions, and soil quality (Rodríguez‐Rodríguez *et al*., 2013). In the present study, the levels of seed oil production and overall plant growth for Camelina transgenics and WT plants were determined from plants grown in pots under glasshouse conditions. The variations between individual plants could be due to the variations in growth conditions, that is soil water content and nutrient levels. We observed consistent results obtained from T3 and T4 homozygous seed generations of plants grown under similar controlled conditions and using random block design experiment.

The percentage of seed oil content of WT plants was about 28.5% on average, which was determined using the NMR spectroscopy. This obtained percentage of seed oil coincides with the previous reports where Camelina seed oil was quantified as the total FAME content, using completely different parameters (Kim *et al*., 2016; Li *et al*., 2015; Rodríguez‐Rodríguez *et al*., 2013).

In a survey conducted in 2007, it was reported that the percentage of oil content of various oilseeds crops is ranging from 20% to 50%, with the oil fruit coconut accumulating the highest amount (65%). The transgenic approach appears to be a positive strategy to boost seed oil content to the upper limit, which still not known in many of the oilseed crops, including Camelina (Taylor *et al*., 2009). By manipulating the expression of DGAT1 and GPD1 in the present study, we were able to increase the seed oil content from 28.5% in WT to 33% in the transgenics, on an average basis (see Table S1). The highest increase in seed mass, seed yield, oil contents and total oil yields was observed in the lines co‐expressing the native DGAT1 with GPD1 (GPD1 + DGAT1 #12 and #17). These Camelina plants can produce an average of ~11.0 g/plant seed yield and 3.8 g/plant oil yield, when grown under glasshouse conditions, thus exhibiting the highest harvest index (Table S1). This reflects their ability to convert photosynthesized products into an economically valuable form, that is seed oil content, under the applied growth conditions. However, it remains to be established whether a similar improvement in seed and oil qualities can be achieved in the natural field conditions.

Therefore, to boost seed oil content in Camelina to the upper limit, we suggest that it may be helpful to combine overexpression of multiple enzymes to further improve seed and oil yields in Camelina and other commercial oilseed crops although it is not quite obvious that this combination approach would be feasible and efficient in other oil seed crops. The levels of seed oils in Camelina or other oilseed crops could be further increased if the factors that limit the production of oils in the developing seeds are illustrated. It was reported that the seed oil content is controlled by several constraints, including the bottlenecks in the TAG metabolic pathways, the oxygen and light availability in seed tissues, and the developmental regulation of TAG biosynthesis (Abdullah *et al*., 2016; Baud and Lepiniec, 2010). To further increase the seed oil content in Camelina, we need to identify the rate‐limiting step(s) that affect the TAG synthesis and accumulation in seeds. To this endeavour, our future studies will focus on to carry out comprehensive RNA‐Seq, metabolome and lipidome analysis of the Camelina transgenics co‐expressing GDP1 and DGAT1 in comparison with WT. By integrating these ‘omics’ approaches, we will be able to identify the bottlenecks that can be targeted to increase the TAG accumulation further.

## Experimental procedures

### Plant material

*Camelina sativa* (L.) Crantz cultivar ‘Suneson’ was grown in the glasshouse at 22°C under natural light conditions supplemented with high‐pressure sodium lights (light threshold of 566 μmol/m^2^/s) with a 16‐h photoperiod (16 h of light and 8 h of darkness), and a 50% minimum humidity. Plants were watered regularly and were fertilized with 200 ppm N of Peters Professional 20‐10‐20 Peat‐lite water‐soluble fertilizer.

### Generation of GmGly::DGAT1, GmGly::DGAT1S205A and GmOle::GPD1 constructs for plant transformation

To overexpress Arabidopsis *DGAT1* and Yeast *GPD1* genes into Camelina seeds, the coding sequences for *DGAT1* (TAIR ID: At3g54320) and *GPD1* (GenBank ID: AY598965) were synthesized using codon‐optimized genes for expression in Camelina by GenScript (GenScript, Piscataway NJ, http://www.genscript.com/). The *glycinin* promoter from soybean (*Glycine max*) was selected to drive the expression of *DGAT1* (Fatihi *et al*., 2013), and the *oleosin* promoter from soybean was chosen to drive the expression of *GPD1*. To test whether a mutant DGAT1 can have an additive effect on seed oil content, a phosphorylation site was abolished by changing serine^205^ to alanine for increased DGAT1 activity as per Xu *et al*. (2008). The three individual gene constructs, GmGly::DGAT1 (referred here as DGAT1), GmGly::DGAT1S205A (referred here as DGAT1 m) and GmOle::GPD1 (referred here as GPD1), were cloned into a multisite Gateway system (Invitrogen, Carlsbad) and then transferred into a binary destination pBnRGW vector, which has DsRed fluorescence marker driven by *Napin* promoter (Figure 1). The sequences of the constructs were verified before they were introduced into the *Agrobacterium tumefaciens* strain GV3101. Six‐week‐old Camelina plants were transformed using the floral dip method (Lu and Kang, 2008). For cotransformation, cultures of *Agrobacterium* containing DGAT1 and those containing GPD1 constructs were blended in equivalent concentrations and used to transform Camelina. T1 seed generation of the transgenic plants were harvested and screened for the expression of DsRed, in which the seeds were illuminated by a green LED flashlight attached to a DsRed filter (Pearstone Inc.) as previously described (Lu and Kang, 2008). T1 transgenic plants were further screened for the expression of the herbicide‐resistant *bar* gene by spraying seedlings grown in soil‐filled pots with the herbicide Basta (containing phosphinothricin) and observed over a 1‐week period for survival as by Kang *et al*. (2011).

### Genotyping of transgenic plants by PCR and confirmation of gene expression using qRT‐PCR

To verify the integration of the transgenes into the genomes of the transgenic plants, PCR was performed on genomic DNA obtained from leaves of selected T1 plants using the standard CTAB protocol, and the presence of promoter‐transgene fragments was confirmed. To investigate the expression of the transgenes, total RNA was extracted from developing seeds at 16–21 days after flowering (DAF) of transgenics and WT plants using Spectrum Plant Total RNA kit (Sigma–Aldrich) following manufacturer's instructions. RNA concentrations were measured in ng/μL, and purity ratios (260/280 nm and 260/230 nm) were calculated using NanoDrop 2000 spectrophotometer (Thermo Scientific) following manufacturer's instructions. The cDNA pools were quantified and then diluted to a final concentration of 100 ng/μL and were used as templates for qRT‐PCR. Camelina *Glyceraldehyde‐3‐phosphate dehydrogenase* (*GAPDH*) was used as an internal reference in comparisons of gene expression data. All qRT‐PCRs were performed in Eppendorf Mastercycler^®^ep realplex thermal cycler using the intercalation dye ABsolute Blue qPCR SYBR Green master mix kit (Thermo Scientific) as a fluorescent reporter. All PCRs were performed in triplicate. The cDNAs were amplified, and PCR products were quantified using the 2^−ΔΔCt^ method (Schmittgen and Livak, 2008). The error bars represent the standard errors for the fold changes of relative gene expression. WT samples were used as calibrators. The PCR primers for genes and promoter fragments are summarized in Table S2.

### Oil content determinations and FAME analysis

Fatty acid methyl esters (FAMEs) were prepared from Camelina mature seeds of transgenics and WT plants according to the Kansas Lipidomics Research Center standard protocols, and the method modified from Iven *et al*. (2013). The extracted FAMEs were quantified using 6890N GC (Agilent Technologies) coupled to a flame ionization detector (FID) on HP‐88 capillary column (column length—100 m, internal diameter—250 μm, film thickness—0.20 μm) with helium as a carrier gas. Seed oil content was measured by the low‐resolution time domain NMR spectroscopy using a Burker Minispec MQ20 (Brurker Optik GmbH, 76275 Ettlingen, Germany). The oil calibration was constructed according to the manufacturer's instructions using pure Camelina oil. The total oil yield was calculated by multiplying oil content and seed yield, and the values were expressed as gram oil per plant. The oil harvest index was expressed as mg oil per mg of total plant dry matter as described by (Li *et al*., 2015). At maturation, seed pods were harvested, and then the plant biomass was dried and weighted, and harvest index was determined according to the formula: Harvest index = (economical yield/biological yield) × 100 (Vafaei *et al*., 2010). Samples were analysed in triplicates unless otherwise mentioned.

### Seed attributes analysis

The seeds were harvested when plants matured and the seed pods were dry. The seeds were cleaned using a sieve and dried at room temperature for a week. Seeds harvested from each plant were weighed to determine the seed yield. The weight of 100 Camelina seeds was used to indicate the seed mass. For total plant biomass measurements, the shoots and roots, after seed harvesting, were dried at 60°C for 14 days before weighing.

### Protein content determination in Camelina seeds

Total protein content in seeds was measured according to (Vigeolas *et al*., 2007). Briefly, 10 mg Camelina seeds from WT and transgenic plants were homogenized in 1 mL of 50 mm 4‐(2‐hydroxyethyl)‐1‐piperazineethanesulfonic acid (HEPES)/NaOH, pH 7.4, using a polytron. The protein content in the homogenate was quantified using the dye‐binding assay (Bradford, 1976) with bovine serum albumin (BSA) as the standard.

### Seedling growth assay

A total of 100 Camelina seeds were germinated over moist papers containing enough moisture in Petri plates. The plates were then placed in a growth chamber set at 24°C, and the moisture was maintained. The weights of 5‐day‐old seedlings were measured from the germinated seedlings, and the data were recorded as mg per plant. The measurements were done in triplicates for each genotype.

### Statistical analysis

The number of replicates (*n*) and the standard error (SE) are shown for most measurements. The seed and oil quality data were analysed with the SAS version 9.1(www.sas.com)using ANOVA (*P* < 0.05) on the corresponding degrees of freedom (df), followed by Dunnett's procedure for pairwise comparisons of all treatments to an untransformed WT control. The authors declare no conflict of interest.

## Authors’ contributions

OPD and DS conceived the study and oversaw its design and coordination. SC and HMA performed the experiments and collected and analysed the data. PA helped with growing plants and harvesting tissue samples. HMA and OPD drafted the manuscript. All authors have been involved in revising the manuscript critically for important intellectual contents. All authors have read and approved the final manuscript.

## Supporting information


**Figure S1** PCR genotyping of T1 generation developing seeds to confirm the integration of transgenes.
**Figure S2** Individual and combined effects of GPD1 and DGAT1 expression on seed germination rate after 14 h (A) and early seedling growth rate at 38 h (B).
**Table S1** Seed attributes (seed yield, seeds mass, %oil contents, oil yield oil harvest index, seed husk weight, and plant biomass) of Camelina transgenic lines.
**Table S2** PCR primers designed to investigate integration and expression of the transgenes into transgenic Camelina seeds.
