# Supplementary material for: Engineering Camelina sativa (L.) Crantz for enhanced oil and seed yields by combining diacylglycerol acyltransferase1 and glycerol‐3‐phosphate dehydrogenase expression
Source: Plant Biotechnol J. 2017 Nov 19;16(5):1034–45. doi: 10.1111/pbi.12847 (PMC5902773; doi:10.1111/pbi.12847)
Supplement: Supplementary file 1 — Figure S1 PCR genotyping of T1 generation developing seeds to confirm the integration of transgenes. Figure S2 Individual and combined effects of GPD1 and DGAT1 expression on seed germination rate after 14 h (A) and early seedling growth rate at 38 h (B). Table S1 Seed attributes (seed yield, seeds mass, %oil contents, oil yield oil harvest index, seed husk weight, and plant biomass) of Camelina transgenic lines. Table S2 PCR primers designed to investigate integration and expression of the transgenes into transgenic Camelina seeds. [file PBI-16-1034-s001.docx]

**Engineering *Camelina sativa* (L.) Crantz for Enhanced Oil and Seed Yields by Combining Diacylglycerol Acyltransferase1 and Glycerol-3-Phosphate Dehydrogenase Expression**

^¥^ Sudesh Chhikara^1,a^, ^¥^ Hesham M. Abdullah^1,2^, Parisa Akbari^1^, Danny Schnell^3^, and Om Parkash Dhankher^1*^

^1^Stockbridge School of Agriculture, University of Massachusetts Amherst, Amherst, MA 01003, USA;

^2^Biotechnology Department, Faculty of Agriculture, Al-Azhar University, Cairo 11651, Egypt

^3^Department of Plant Biology, Michigan State University, East Lansing, MI 48824, USA.

^¥^ These authors are contributed equally to this work

^a^Present address: Centre for Biotechnology, Maharshi Dayanand University, Rohtak 124001, India

^*^**Corresponding author**: Om Parkash Dhankher; email: [parkash@umass.edu](mailto:parkash@umass.edu)

Emails:

Sudesh Chhikara: sushchhikara@gmail.com

Hesham M. Abdullah: [habdullah@psis.umass.edu](mailto:habdullah@psis.umass.edu)

Parisa Akbari: [pakbari@psis.umass.edu](mailto:pakbari@psis.umass.edu)

Danny Schnell: [dschnell@cns.msu.edu](mailto:dschnell@cns.msu.edu)

Om Parkash Dhankher: [**parkash@umass.edu**](mailto:parkash@umass.edu)

| Table S1. Seed attributes (seed yield, seeds mass, %oil contents, oil yield, oil harvest index, seed husk weight, and plant biomass) of Camelina transgenic lines. | | | | | | | | | | | | | |
| --- | --- | --- | --- | --- | --- | --- | --- | --- | --- | --- | --- | --- | --- |
| Constructs | **Seed yield (g/plant)** | **Seed weight (g/100 seeds)** | | | **Gain in seed weight relative to WT (%)** | **Seed husk weight (g/plant)** | **Total plant biomass (g/plant)** | **Oil %**  **(wt/wt)** | | | **Gain in oil content relative to WT (%)** | **Oil yield**  **(g/plant)** | **Harvest index** |
| WT | 5.05 | 0.095 | | ± 0.0049476 | - |  | 13.83 | 28.58 |  | ± 0.26 | - | 1.44 | 0.105 |
| GPD1 lines | | | | | | | | | | | | | |
| 8-4-2 | 4.54 | 0.123 |  | ± 0.0014142 | 29.4% | 2.7 | 11.64 | 30.58 |  | ± 0.28 | 7.0% | 1.388332 | 0.119273 |
| 8-4-3 | 4.75 | 0.1175 |  | ± 0.0005774 | 23.7% | 2.5 | 11.45 | 30.87 |  | ± 0.22 | 8.0% | 1.466325 | 0.128063 |
| 8-4-4 | 5.9 | 0.11925 |  | ± 0.0005 | 25.5% | 3.9 | 16.6 | 30.8 |  | ± 0.19 | 7.8% | 1.8172 | 0.10947 |
| 8-4-5 | 7.16 | 0.125 |  | ± 0.0011547 | 31.5% | 3.7 | 16.56 | 32.96 |  | ± 0.28 | 15.3% | 2.359936 | 0.142508 |
| 8-4-6 | 5.04 | 0.1375 |  | ± 0.001291 | 44.7% | 2.8 | 13.24 | 32.88 |  | ± 0.39 | 15.0% | 1.657152 | 0.125163 |
| 8-4-7 | 6.35 | 0.12925 |  | ± 0.0005 | 36.0% | 4.6 | 16.15 | 31.06 |  | ± 0.11 | 8.7% | 1.97231 | 0.122124 |
| 8-4-8 | 6.02 | 0.1185 |  | ± 0.0005774 | 24.7% | 3.3 | 15.92 | 31.25 |  | ± 0.03 | 9.3% | 1.88125 | 0.118169 |
| 8-4-10 | 6.82 | 0.11975 |  | ± 0.0012583 | 26.0% | 5 | 17.02 | 30.13 |  | ± 0.3 | 5.4% | 2.054866 | 0.120732 |
| 15-3-1 | 8.65 | 0.12725 |  | ± 0.0009574 | 33.9% | 4.4 | 20.45 | 31.23 |  | ± 0.2 | 9.3% | 2.701395 | 0.132098 |
| 15-3-2 | 5.97 | 0.11025 |  | ± 0.0005 | 16.0% | 3.3 | 14.87 | 30.1 |  | ± 0.25 | 5.3% | 1.79697 | 0.120845 |
| 15-3-3 | 5.85 | 0.1265 |  | ± 0.001 | 33.1% | 2.8 | 13.85 | 32.23 |  | ± 0.23 | 12.8% | 1.885455 | 0.136134 |
| 15-3-4 | 6.88 | 0.112 |  | ± 0.0008165 | 17.9% | 3.9 | 17.58 | 29.34 |  | ± 0.19 | 2.7% | 2.018592 | 0.114823 |
| 15-3-7 | 5.42 | 0.11525 |  | ± 0.0005 | 21.3% | 2.7 | 13.32 | 29.9 |  | ± 0.22 | 4.6% | 1.62058 | 0.121665 |
| 15-3-8 | 4.87 | 0.13225 |  | ± 0.0025 | 39.2% | 2.8 | 14.27 | 29.49 |  | ± 0.07 | 3.2% | 1.436163 | 0.100642 |
| 15-3-9 | 8.88 | 0.12325 |  | ± 0.0012583 | 29.7% | 4.5 | 20.38 | 30.5 |  | ± 0.21 | 6.7% | 2.7084 | 0.132895 |
| 15-3-10 | 5.26 | 0.11975 |  | ± 0.0005 | 26.0% | 2.7 | 13.16 | 31.5 |  | ± 0.27 | 10.2% | 1.6569 | 0.125904 |
| 15-12-1 | 7.67 | 0.11975 |  | ± 0.0017078 | 26.0% | 3.8 | 18.77 | 30.25 |  | ± 0.24 | 5.8% | 2.320175 | 0.123611 |
| 15-12-2 | 6.59 | 0.1265 |  | ± 0.0005774 | 33.1% | 2.9 | 15.69 | 30.83 |  | ± 0.16 | 7.8% | 2.031697 | 0.12949 |
| 15-12-3 | 6.08 | 0.11975 |  | ± 0.0005 | 26.0% | 3.1 | 14.78 | 30.75 |  | ± 0.15 | 7.6% | 1.8696 | 0.126495 |
| 15-12-6 | 6.79 | 0.131 |  | ± 0.0014142 | 37.9% | 3.8 | 17.49 | 31.09 |  | ± 0.18 | 8.8% | 2.111011 | 0.120698 |
| 15-12-7 | 7.4 | 0.11825 |  | ± 0.0012583 | 24.4% | 3.8 | 17.7 | 29.33 |  | ± 0.29 | 2.6% | 2.17042 | 0.122623 |
| 15-12-8 | 5.41 | 0.1145 |  | ± 0.0005774 | 20.5% | 3 | 14.71 | 29.59 |  | ± 0.45 | 3.5% | 1.600819 | 0.108825 |
| 15-12-9 | 8.63 | 0.11775 |  | ± 0.0009574 | 23.9% | 5.5 | 23.03 | 29.16 |  | ± 0.2 | 2.0% | 2.516508 | 0.109271 |
| DGAT1m lines | | | | | | | | | | | | | |
| 2-1-1 | 4.73 | 0.113625 |  | ± 0.0009946 | 19.6% | 2.6 | 12.93 | 29.19 |  | ± 0.17 | 2.1% | 1.380687 | 0.106782 |
| 2-1-3 | 4.42 | 0.115875 |  | ± 0.0024061 | 21.9% | 2.2 | 10.92 | 30.17 |  | ± 0.09 | 5.6% | 1.333514 | 0.122117 |
| 2-1-4 | 6.18 | 0.11635 |  | ± 0.003851 | 22.4% | 3 | 15.98 | 30.52 |  | ± 0.14 | 6.8% | 1.886136 | 0.118031 |
| 2-1-5 | 5.92 | 0.116925 |  | ± 0.0008057 | 23.0% | 3.3 | 15.82 | 29.75 |  | ± 0.21 | 4.1% | 1.7612 | 0.111327 |
| 2-1-7 | 7.27 | 0.1265 |  | ± 0.0013089 | 33.1% | 3.1 | 17.67 | 31.25 |  | ± 0.26 | 9.3% | 2.271875 | 0.128572 |
| 2-7-1 | 5.8 | 0.10815 |  | ± 0.0032182 | 13.8% | 3 | 14.3 | 29.37 |  | ± 0.57 | 2.8% | 1.70346 | 0.119123 |
| 2-7-4 | 10.04 | 0.1165 |  | ± 0.0005774 | 22.6% | 5.7 | 24.64 | 30.3 |  | ± 0.2 | 6.0% | 3.04212 | 0.123463 |
| 2-7-5 | 5.75 | 0.11415 |  | ± 0.0022869 | 20.1% | 2.8 | 14.95 | 29.87 |  | ± 0.07 | 4.5% | 1.717525 | 0.114885 |
| 2-7-6 | 9.11 | 0.119025 |  | ± 0.0011354 | 25.3% | 4.8 | 22.41 | 29.21 |  | ± 0.13 | 2.2% | 2.661031 | 0.118743 |
| 33-14-1 | 6.34 | 0.116475 |  | ± 0.0002986 | 22.6% | 3.5 | 15.94 | 29.5 |  | ± 0.17 | 3.2% | 1.8703 | 0.117334 |
| 33-14-2 | 4.08 | 0.107425 |  | ± 0.0012842 | 13.0% | 2.5 | 11.28 | 29.4 |  | ± 0.17 | 2.9% | 1.19952 | 0.10634 |
| 33-14-3 | 6.64 | 0.117425 |  | ± 0.0013574 | 23.6% | 3.5 | 16.14 | 31.62 |  | ± 0.33 | 10.6% | 2.099568 | 0.130085 |
| 33-14-4 | 6.11 | 0.1171 |  | ± 0.002459 | 23.2% | 3.4 | 15.71 | 30.72 |  | ± 0.14 | 7.5% | 1.876992 | 0.119478 |
| 33-14-5 | 4.75 | 0.109875 |  | ± 0.0042217 | 15.6% | 2.5 | 11.55 | 29.4 |  | ± 0.09 | 2.9% | 1.3965 | 0.120909 |
| 33-14-7 | 9.8 | 0.110375 |  | ± 0.0016581 | 16.2% | 5.6 | 24.3 | 29.37 |  | ± 0.16 | 2.8% | 2.87826 | 0.118447 |
| 33-14-9 | 5.35 | 0.1101 |  | ± 0.003747 | 15.9% | 2.8 | 13.15 | 30.18 |  | ± 0.11 | 5.6% | 1.61463 | 0.122786 |
| 33-15-3 | 5.21 | 0.114525 |  | ± 0.0020023 | 20.5% | 2.6 | 12.71 | 30.33 |  | ± 0.2 | 6.1% | 1.580193 | 0.124327 |
| 33-15-4 | 7.94 | 0.10305 |  | ± 0.0014387 | 8.4% | 4.8 | 19.74 | 32.1 |  | ± 0.07 | 12.3% | 2.54874 | 0.129116 |
| 33-15-5 | 4.39 | 0.12095 |  | ± 0.0013699 | 27.3% | 2.5 | 11.29 | 31.65 |  | ± 0.07 | 10.7% | 1.389435 | 0.123068 |
| 33-15-6 | 8.07 | 0.1204 |  | ± 0.0011576 | 26.7% | 4.6 | 19.37 | 31.58 |  | ± 0.11 | 10.5% | 2.548506 | 0.13157 |
| 33-15-9 | 6.55 | 0.114875 |  | ± 0.0012366 | 20.9% | 3.8 | 16.65 | 30.02 |  | ± 0.23 | 5.0% | 1.96631 | 0.118097 |
| 33-15-10 | 3.99 | 0.110125 |  | ± 0.0013598 | 15.9% | 2.2 | 11.19 | 29.67 |  | ± 0.15 | 3.8% | 1.183833 | 0.105794 |
| DGAT1 lines | | | | | | | | | | | | | |
| 4-7-3 | 6.83 | 0.11085 |  | ± 0.0010017 | 16.7% | 3.4 | 17.23 | 29.22 |  | ± 0.15 | 2.2% | 1.995726 | 0.115829 |
| 4-7-4 | 5.69 | 0.11545 |  | ± 0.0007681 | 21.5% | 3.4 | 16.19 | 29.25 |  | ± 0.13 | 2.3% | 1.664325 | 0.1028 |
| 4-7-5 | 5.95 | 0.108225 |  | ± 0.0008016 | 13.9% | 4 | 16.55 | 29.09 |  | ± 0.12 | 1.8% | 1.730855 | 0.104583 |
| 4-7-6 | 9.89 | 0.11155 |  | ± 0.0008021 | 17.4% | 5.8 | 24.19 | 30.27 |  | ± 0.16 | 5.9% | 2.993703 | 0.123758 |
| 4-7-8 | 8.21 | 0.119225 |  | ± 0.0011587 | 25.5% | 5.4 | 21.91 | 29.47 |  | ± 0.04 | 3.1% | 2.419487 | 0.110428 |
| 4-7-9 | 8.12 | 0.113025 |  | ± 0.0034053 | 18.9% | 4.1 | 19.42 | 29.81 |  | ± 0.14 | 4.3% | 2.420572 | 0.124643 |
| 12-3-5 | 6.19 | 0.1195 |  | ± 0.0010231 | 25.8% | 3.6 | 16.49 | 30.53 |  | ± 0.13 | 6.8% | 1.889807 | 0.114603 |
| 12-3-4 | 4.56 | 0.0914 |  | ± 0.0022672 | -3.8% | 2.8 | 13.16 | 31.974 |  | ± 0.12 | 12.0% | 2.38238 | 0.125338 |
| 12-3-9 | 5.5 | 0.1041 |  | ± 0.0010145 | 9.6% | 4.2 | 16.3 | 31.594 |  | ± 0.16 | 11.0% | 2.861479 | 0.167435 |
| GPD1 + DGAT1 lines | | | | | | | | | | | | | |
| 12-3-3 | 6.95 | 0.145175 |  | ± 0.002278 | 57.2% | 4.8 | 12.9 | 30.62 |  | ± 0.35 | 7.1% | 2.12809 | 0.164968 |
| 12-3-8 | 7.73 | 0.133 |  | ± 0.0031591 | 44.0% | 5.2 | 14.5 | 31.74 |  | ± 0.1 | 11.0% | 2.453502 | 0.169207 |
| 12-3-11 | 11.29 | 0.12855 |  | ± 0.0011358 | 39.2% | 5.8 | 16.3 | 34.51 |  | ± 0.31 | 20.7% | 3.896179 | 0.239029 |
| 12-3-14 | 7.15 | 0.1368 |  | ± 0.0029833 | 48.2% | 3.3 | 10 | 33.32 |  | ± 0.13 | 16.5% | 2.38238 | 0.238238 |
| 12-3-25 | 10.62 | 0.155025 |  | ± 0.0017095 | 67.9% | 7.5 | 19.9 | 30.08 |  | ± 0.06 | 9.5% | 3.194496 | 0.160527 |
| 12-3-29 | 13.2 | 0.149325 |  | ± 0.0014908 | 61.7% | 7.9 | 21.1 | 31.03 |  | ± 0.55 | 8.5% | 4.09596 | 0.194121 |
| 12-3-30 | 9.2 | 0.154125 |  | ± 0.0002986 | 66.9% | 5.9 | 15.1 | 31.53 |  | ± 0.11 | 10.3% | 2.90076 | 0.192103 |
| 17-17-4 | 7.51 | 0.141625 |  | ± 0.001537 | 53.4% | 3.6 | 11 | 32.57 |  | ± 0.11 | 14.0% | 2.446007 | 0.222364 |
| 17-17-6 | 8.22 | 0.120925 |  | ± 0.0022142 | 31.0% | 4.6 | 12 | 30.96 |  | ± 0.2 | 8.3% | 2.544912 | 0.212076 |
| 17-17-7 | 12.63 | 0.133725 |  | ± 0.0012198 | 44.8% | 5.9 | 15.9 | 32.65 |  | ± 0.19 | 14.2% | 4.123695 | 0.259352 |
| 17-17-8 | 8.06 | 0.13615 |  | ± 0.001245 | 47.4% | 4 | 12.1 | 31 |  | ± 0.18 | 8.5% | 2.4986 | 0.206496 |
| 17-17-9 | 11.05 | 0.1305 |  | ± 0.0011314 | 41.3% | 5.1 | 14.9 | 33.91 |  | ± 0.2 | 18.6% | 3.747055 | 0.25148 |
| 17-17-10 | 7.17 | 0.13925 |  | ± 0.002541 | 50.8% | 3.4 | 10.1 | 32.75 |  | ± 0.32 | 14.6% | 2.348175 | 0.232493 |
| 17-17-13 | 10.83 | 0.136225 |  | ± 0.0022765 | 47.5% | 5.5 | 14.8 | 31.8 |  | ± 0.18 | 11.3% | 3.44394 | 0.232699 |
| 17-17-14 | 10.89 | 0.134275 |  | ± 0.0004419 | 45.4% | 5.4 | 15.6 | 33.6 |  | ± 0.28 | 17.6% | 3.65904 | 0.234554 |
| GPD1 + DGAT1m lines | | | | | | | | | | | | | |
| 11-16-1 | 7.75 | 0.1285 |  | ± 0.0006 | 39.2% | 3.7 | 10.6 | 32.35 |  | ± 0.1 | 13.2% | 2.507125 | 0.236521 |
| 11-16-4 | 10.21 | 0.129025 |  | ± 0.0007248 | 39.7% | 5.8 | 15.6 | 32.14 |  | ± 0.1 | 12.5% | 3.281494 | 0.210352 |
| 11-16-5 | 10.25 | 0.1289 |  | ± 0.0006218 | 39.6% | 3.5 | 10.3 | 32.18 |  | ± 0.04 | 12.6% | 3.29845 | 0.320238 |
| 11-16-10 | 10.44 | 0.133125 |  | ± 0.0015756 | 44.2% | 5.4 | 15.8 | 32.08 |  | ± 0.23 | 12.2% | 3.349152 | 0.211972 |
| 11-16-11 | 9.69 | 0.11985 |  | ± 0.0001768 | 29.8% | 5.2 | 14.9 | 32.38 |  | ± 0.23 | 13.3% | 3.137622 | 0.210579 |
| 11-16-12 | 11.26 | 0.13045 |  | ± 0.0014248 | 41.3% | 5.5 | 16 | 32.31 |  | ± 0.44 | 13.1% | 3.638106 | 0.227382 |
| 11-16-14 | 9.55 | 0.13475 |  | ± 0.0007425 | 45.9% | 5.1 | 14.6 | 31.25 |  | ± 0.2 | 9.3% | 2.984375 | 0.204409 |
| 11-16-15 | 6.68 | 0.1284 |  | ± 0.0005477 | 39.1% | 5 | 14.8 | 32.16 |  | ± 0.14 | 12.5% | 2.148288 | 0.145155 |
| 9-25-0 | 5.80 | 0.124 |  | ± 0.0005678 | 30.6% | 3.6 | 10.3 | 31.72 |  | ± 0.17 | 11.0% | 1.83976 | 0.178617476 |
| 9-30-0 | 4.511 | 0.124 |  | ± 0.0009844 | 30.3% | 2.224 | 6.924 | 31.461 |  | ± 0.217 | 10.1% | 1.41920571 | 0.204969051 |
| 9-31-0 | 4.952 | 0.125 |  | ± 0.0001290 | 31.4% | 2.637 | 7.537 | 31.229 |  | ± 0.745 | 9.3% | 1.54646008 | 0.205182444 |
| 9-32-0 | 11.65 | 0.125 |  | ± 0.0001290 | 31.8% | 6.522 | 17.522 | 31.608 |  | ± 0.128 | 10.6% | 3.682332 | 0.210154777 |
| 9-33-0 | 7.718 | 0.126 |  | ± 0.0001290 | 32.2% | 3.443 | 10.943 | 32.514 |  | ± 0.359 | 13.8% | 2.50943052 | 0.229318333 |
| 9-34-0 | 10.957 | 0.126 |  | ± 0.0001290 | 32.6% | 6.131 | 16.831 | 31.541 |  | ± 0.099 | 10.4% | 3.45594737 | 0.205332266 |
| 9-35-0 | 6.359 | 0.128 |  | ± 0.0005066 | 34.6% | 3.241 | 8.741 | 31.802 |  | ± 0.344 | 11.3% | 2.02228918 | 0.23135673 |
| 9-36-0 | 6.124 | 0.135 |  | ± 0.0003366 | 42.4% | 3.287 | 9.487 | 31.886 |  | ± 0.258 | 11.6% | 1.95269864 | 0.205828886 |
| 9-37-0 | 5.739 | 0.136 |  | ± 0.0025011 | 43.4% | 2.504 | 8.204 | 32.094 |  | ± 0.193 | 12.3% | 1.84187466 | 0.224509344 |
| 9-38-0 | 8.23 | 0.129 |  | ± 0.0004203 | 35.9% | 4.317 | 12.417 | 32.698 |  | ± 0.148 | 14.4% | 2.6910454 | 0.216722671 |
| *Percent weight gain, % oil content, and seed and biomass yields in transformed T3 homozygous lines as compared to their relative wildtype (WT) as a control. The values in seed yield, seed husk weight, plant biomass, oil yield, and harvest index are the exact values measured in individual plants while the values in seed weight, Oil% represent the mean ± standard deviation from at least three independent measurements.* | | | | | | | | | | | | | |

| Table S2. PCR primers designed to investigate integration and expression of the transgenes into Camelina seeds of transgenic lines | | |
| --- | --- | --- |
| Primer name | Target gene/promoter | Forward/Reverse primers |
| OleMet | Glycine max oleosin promoter | 5’TACGTCGGGCCCGTACGTAGTGTTTATCTTTGTTGCTTTTCT3’  5’TAGCTGGTCGACCCATGGGCGGCCGCGGTTGAAGGTGAAGTT TA3’ |
| GmGly | Glycine max glycinin promoter | 5’TACGTCGGGCCCAAGCTTTAGCCT AAG TAC GTA CTC AAA ATG CCA A 3’ 5’- TAG CTG GTC GAC GGA TCC TCT AGA CAT ATG CCA TGGGGTGATGACTGATGAGTG TTT AAG GA 3’ |
| GDP1S | Glycerol-3-phosphate dehydrogenase | 5’- TAC GTC CCA TGG ATG TCT GCG GCT GCG GAT AGA TTA 3’  5’- TAG CTG AAG CTT GTC GAC GAA TTC TCA CAG ATC TTC TTC AGA GAT CAG TTT CTG TTC GTC TTC AT 3’ |
| DGAT1S | Diacylglycerol acyltransferase | 5’- TAC GTC CCA TGG CAT ATG GCC ATA TTA GAC TCG GCA GGG GT 3’  5’- TAG CTG GGA TCC TCT AGA TCA CAG ATC TTC TTC AGA GAT CAG TTT CTG TTC TGA CAT GCT A 3’ |
| GPD1-350 | Glycerol-3-phosphate dehydrogenase | 5’- GAT GTC CAC ATC TTT CAC GGA GTC AAT CA 3’ |
| DGAT1-310 | Diacylglycerol acyltransferase | 5’- TGG CAT CAG CGT TAC CTC TAC CCT CAC CT |


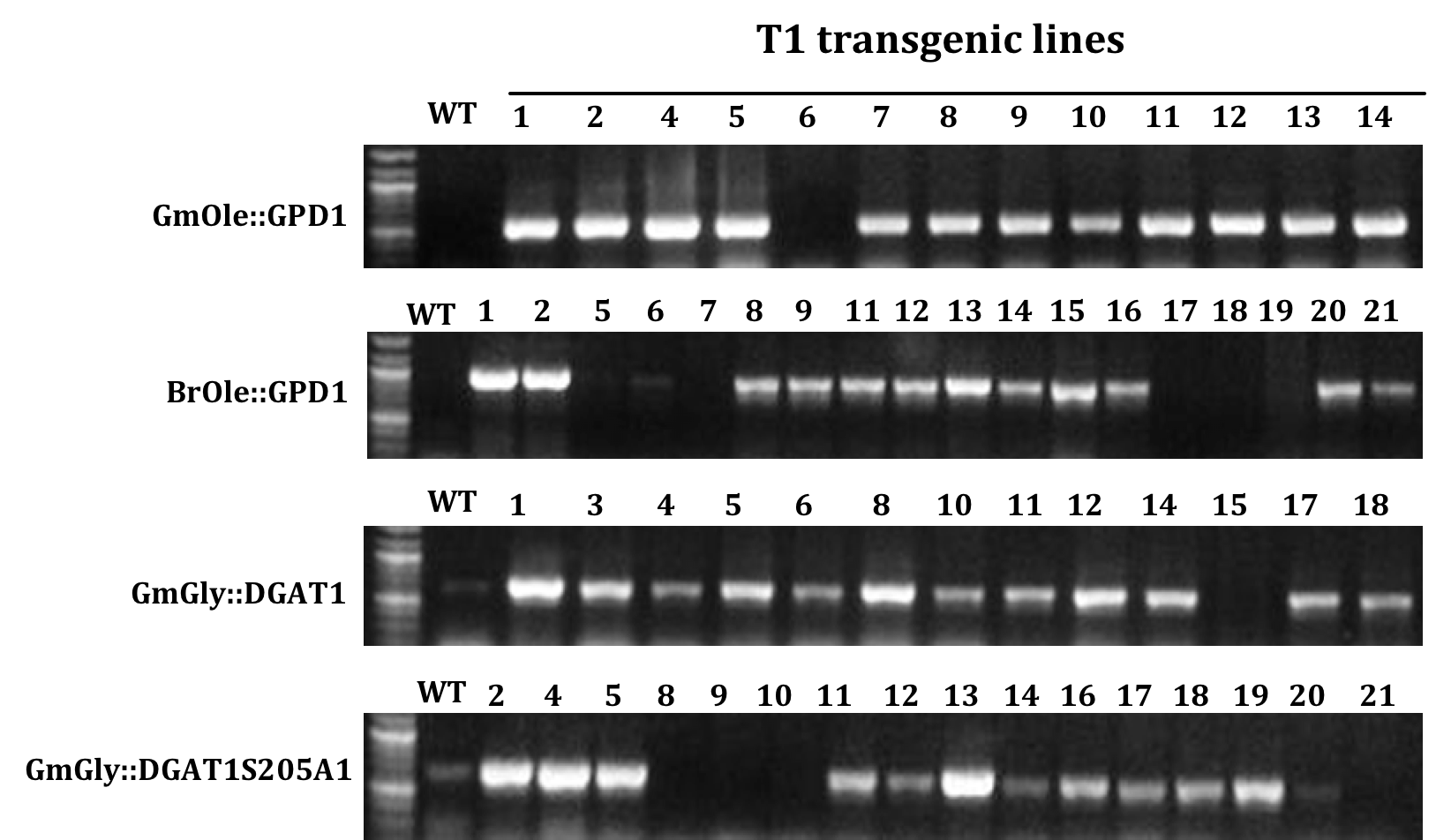


**A**

**GPD1+DGAT1S205A**

**GPD1 primer**

**DGAT1M primer**

**WT**

**9**

**11**

**11**

**WT**

**9**


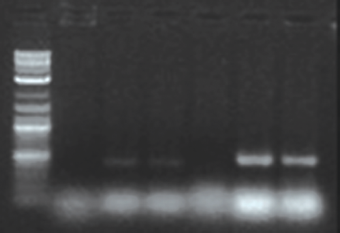


**B**

**GPD1+DGAT1**

**GPD1 primer**

**DGAT1 primer**

**WT**

**12**

**17**

**18**

**29**

**WT**

**12**

**17**

**18**

**29**

**C**

**Figure S1.** PCR genotyping of T1 generation developing seeds to confirm the integration of transgenes. **A.** PCR primers used were designed to detect the presence of Soybean *Oleosin* (*OLE*) and Soybean *glycinin* (*GLY*) promoters in DGAT1 lines. **B. & C.** Primers designed to detect Soybean *oleosin* and *glycinin* promoters in both GPD1+mDGAT1 and GPD1+DGAT1, respectively.


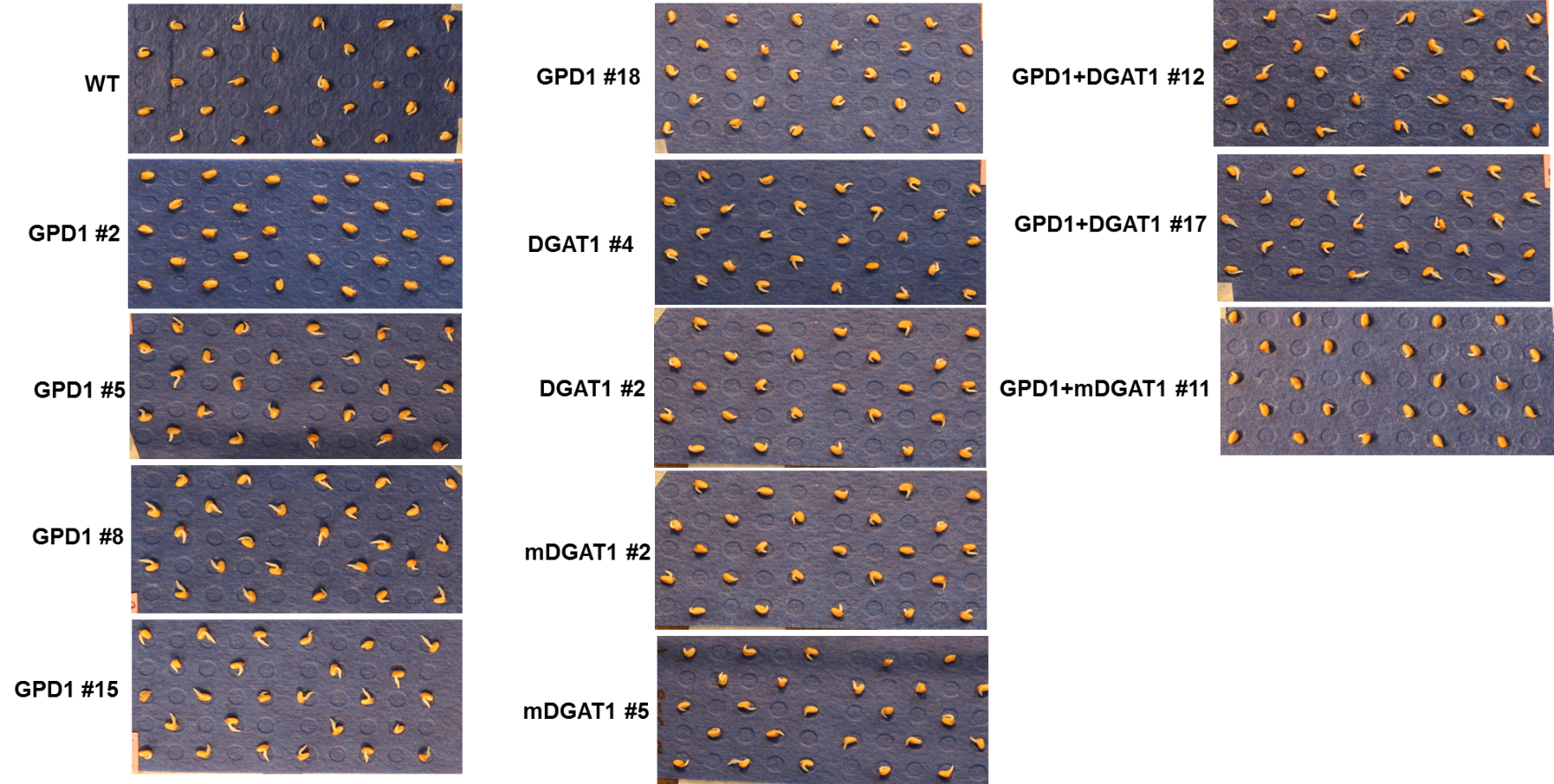


**A**

**B**


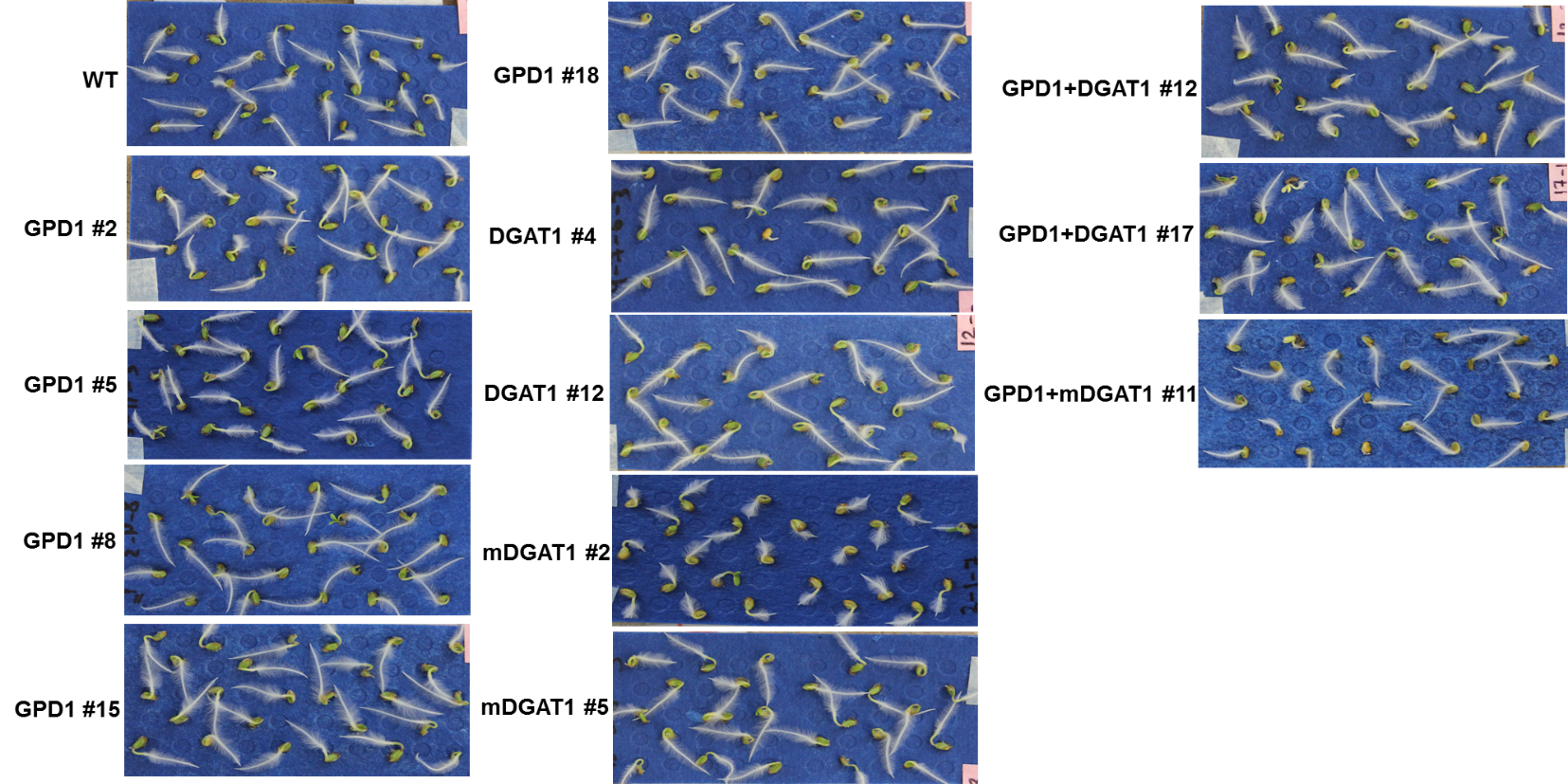


**Figure S2.** Individual and combined effects of GPD1 and DGAT1 expression on seed germination rate after 14 hours **(A)** and early seedling growth rate at 38 hours **(B)**.
